# Inertial bioluminescence rhythms at the Capo Passero (KM3NeT-Italia) site, Central Mediterranean Sea

**DOI:** 10.1038/srep44938

**Published:** 2017-03-23

**Authors:** J. Aguzzi, E. Fanelli, T. Ciuffardi, A. Schirone, J. Craig, KM3NeT-Italia/NEMO Collaboration, S. Aiello, F. Ameli, M. Anghinolfi, G. Barbarino, E. Barbarito, N. Beverini, S. Biagi, A. Biagioni, B. Bouhadef, C. Bozza, G. Cacopardo, M. Calamai, C. Calì, A. Capone, F. Caruso, S. Cecchini, A. Ceres, T. Chiarusi, M. Circella, R. Cocimano, R. Coniglione, M. Costa, G. Cuttone, C. D’Amato, A. D’Amico, G. De Bonis, V. De Luca, N. Deniskina, C. Distefano, L. S. Di Mauro, P. Fermani, G. Ferrara, V. Flaminio, L. A. Fusco, F. Garufi, V. Giordano, A. Gmerk, R. Grasso, G. Grella, C. Hugon, M. Imbesi, V. Kulikovskiy, G. Larosa, D. Lattuada, K. P. Leismüller, E. Leonora, P. Litrico, A. Lonardo, F. Longhitano, D. Lo Presti, E. Maccioni, A. Margiotta, A. Marinelli, A. Martini, R. Masullo, R. Mele, P. Migliozzi, E. Migneco, A. Miraglia, C. M. Mollo, M. Mongelli, M. Morganti, P. Musico, M. Musumeci, C. A. Nicolau, A. Orlando, A. Orzelli, R. Papaleo, C. Pellegrino, M. G. Pellegriti, C. Perrina, P. Piattelli, E. Poma, S. Pulvirenti, F. Raffaelli, N. Randazzo, G. Riccobene, A. Rovelli, M. Sanguineti, P. Sapienza, V. Sciacca, I. Sgura, F. Simeone, V. Sipala, F. Speziale, A. Spitaleri, M. Spurio, S. M. Stellacci, M. Taiuti, G. Terreni, L. Trasatti, A. Trovato, F. Versari, P. Vicini, S. Viola, D. Vivolo

**Affiliations:** 1 Instituto de Ciencias del Mar (ICM) del Consejo Superior de Investigaciones Científicas (CSIC), Paseo Marítimo de la Barceloneta, 37-49, 08003 Barcelona Spain; 2 ENEA, Marine Environment Research Centre, P.O. Box 224, 19100 Pozzuolo di Lerici (SP), Italy; 3 Oceanlab, University of Aberdeen, Main Street, Newburgh, AB41 6AA, UK; 4 INFN Sezione Catania, Via S. Sofia 64, 95123 Catania, Italy; 5 INFN Sezione Roma, P.le A. Moro 2, 00185 Rome, Italy; 6 INFN Sezione Genova, Via Dodecaneso 33, 16146 Genoa, Italy; 7 INFN Sezione Napoli, Via Cintia, 80126 Naples, Italy; 8 Dipartimento di Scienze Fisiche Università di Napoli, Via Cintia, 80126 Naples, Italy; 9 INFN Sezione Bari, Via E. Orabona 4, 70126 Bari, Italy; 10 Dipartimento Interateneo di Fisica Università di Bari, Via E. Orabona 4, 70126 Bari, Italy; 11 INFN Sezione Pisa, Polo Fibonacci, Largo Bruno Pontecorvo 3, 56127 Pisa, Italy; 12 Dipartimento di Fisica Università di Pisa, Polo Fibonacci, Largo Bruno Pontecorvo 3, 56127 Pisa, Italy; 13 INFN Laboratori Nazionali del Sud, Via S.Sofia 62, 95123 Catania, Italy; 14 INFN Gruppo Collegato di Salerno, Via Giovanni Paolo II 132, 84084 Fisciano, Italy; 15 Dipartimento di Fisica Università di Salerno, Via Giovanni Paolo II 132, 84084 Fisciano, Italy; 16 Dipartimento di Fisica Università “Sapienza”, P.le A. Moro 2, 00185 Rome, Italy; 17 INFN Sezione Bologna, V. le Berti Pichat 6/2, 40127 Bologna, Italy; 18 Nikhef, Science Park 105, 1098 XG Amsterdam, The Netherlands; 19 Dipartimento di Fisica ed Astronomia Università di Bologna, V.le Berti Pichat 6/2, 40127 Bologna, Italy; 20 Accademia Navale di Livorno, viale Italia 72, 57100 Livorno, Italy; 21 INFN Laboratori Nazionali di Frascati, Via Enrico Fermi 40, 00044 Frascati, Italy; 22 Dipartimento di Fisica Università di Genova, Via Dodecaneso 33, 16146 Genoa, Italy

## Abstract

In the deep sea, the sense of time is dependent on geophysical fluctuations, such as internal tides and atmospheric-related inertial currents, rather than day-night rhythms. Deep-sea neutrino telescopes instrumented with light detecting Photo-Multiplier Tubes (PMT) can be used to describe the synchronization of bioluminescent activity of abyssopelagic organisms with hydrodynamic cycles. PMT readings at 8 different depths (from 3069 to 3349 m) of the NEMO Phase 2 prototype, deployed offshore Capo Passero (Sicily) at the KM3NeT-Italia site, were used to characterize rhythmic bioluminescence patterns in June 2013, in response to water mass movements. We found a significant (*p* < 0.05) 20.5 h periodicity in the bioluminescence signal, corresponding to inertial fluctuations. Waveform and Fourier analyses of PMT data and tower orientation were carried out to identify phases (i.e. the timing of peaks) by subdividing time series on the length of detected inertial periodicity. A phase overlap between rhythms and cycles suggests a mechanical stimulation of bioluminescence, as organisms carried by currents collide with the telescope infrastructure, resulting in the emission of light. A bathymetric shift in PMT phases indicated that organisms travelled in discontinuous deep-sea undular vortices consisting of chains of inertially pulsating mesoscale cyclones/anticyclones, which to date remain poorly known.

Light as an evolutionary force shapes marine ecosystem functioning, regulating the biological clock of predator and prey species, which react by performing rhythmic displacements into photic and disphotic realms^1^. However, beyond the reach of solar light, where virtually all ecologically-functional radiance is of biogenic origin^2^, the sense of time is dependent upon other geophysical fluctuations such as internal tides and inertial currents^3^,^4^. The deep sea forms the largest biome on Earth, yet the role of these forces on the temporal regulation of water-column and benthic communities’ activity rhythms is to date almost unknown^5^. The behavioural and physiological synchronization of organisms to flow changes^6^ is likely, as currents can form a key selective agent in relation to important ecological functions, such as low-energy dispersal and feeding^7^.

Data on the modulation imposed by cyclic flow changes on the biology of deep-sea organisms, although sparse, suggest the synchronization of behaviour and underlying physiology at internal-tidal periodicities (e.g. refs 6 and 7. However, evidence for the synchronization of biorhythms to the weaker atmospheric-related inertial currents is more scant; e.g. laboratory tests with the deep-water Norway lobster (*Nephrops norvegicus*) and deep-sea crab *Geryon longipes*^8^,^9^,^10^. Inertial currents are generated by wind drift on the water surface producing downward propagating whirlpools, perceived at the seabed as hydrodynamic oscillations. Their periodicity depends on the Coriolis force and therefore on latitude^11^. In the Mediterranean Sea, where tidal forces are very small (except for the North Adriatic)^12^, inertial currents dominate over internal tidal current periodicities, therefore providing a natural laboratory to identify the importance of these motions on marine communities.

Bioluminescence is widespread in the deep sea where it is found in most of the major marine phyla, from bacteria to fish. Luminous signals are an important aspect of deep-sea pelagic and benthic life, where they are used for a wide range of inter-and intraspecific interactions, permitting organisms to find or avoid each other for purposes of defense, offence, and reproduction^2^,^4^,^13^. In undisturbed water, the occurrence of bioluminescent flashes is reported to be rare^14^,^15^, presumably because of the energetic cost of light production^16^. However, defensive bioluminescent responses are provoked when organisms, advected by water currents, collide with submerged objects such as underwater vehicles or complex topography, e.g. rocky outcrops and their protruding sessile fauna^14^,^17^,^18^,^19^. Presently, little is known of the ecological relevance of temporal patterning in the bioluminescence emitted by deep pelagic fauna that results from the interaction of animals with cyclic water flow changes.

Neutrino telescopes deployed in the deep sea, instrumented with light detecting photomultipliers (PMTs) as well as oceanographic and acoustic sensors, are providing data beyond their primary physical purpose and are being used to describe biological phenomena in poorly monitored deep-sea areas. Datasets generated from this technology are enabling new discoveries about life in the abyssopelagic environment^20^,^21^,^22^,^23^ and PMT data are providing evidence of deep blooms in bioluminescence coupled to seasonal changes in passive carbon sinking, linking atmospheric-climatic and demersal components^24^,^25^,^26^.

The Capo Passero site, selected for the installation of the KM3NeT-Italy Neutrino Telescope (http://www.km3net.org^27^ presents appropriate properties for the study of bioluminescence by abyssopelagic organisms and their interaction with water flows of inertial periodicity from the bottom up to few hundreds of meters into the water column. The area is highly oligotrophic^28^,^29^ which results in very low levels of bioluminescent bacteria and bio-fouling on deployed structures at depths greater than 2500 m^30^,^31^. The location of this detector enables data collection on deep layer dynamics of the Eastern basin, which plays an important role in the whole Mediterranean circulation. In this basin, water mass formation and stratification has shown large variability in the last few decades, following the occurrence of the well-known Eastern Mediterranean Transient^32^,^33^,^34^. A recent study by Rubino *el al*.^35^ has shown that abyssal *quasi*-inertial vortices, with mesoscale feature, exist at this site, below 2500 m.

Here, we used high-frequency data retrieved from a prototype detection unit for an underwater neutrino telescope, with two different objectives. An ecological objective was to characterise rhythms in bioluminescence at the different depths of the telescope structure, as a proxy of the benthopelagic movement of organisms within the water column, relative to the seabed. Further, we observed how deep-sea mesoscale vortices may affect bioluminescence rhythms at different depths, generated by organisms in response to the flow of water masses.

## Results

**Figure 1:**
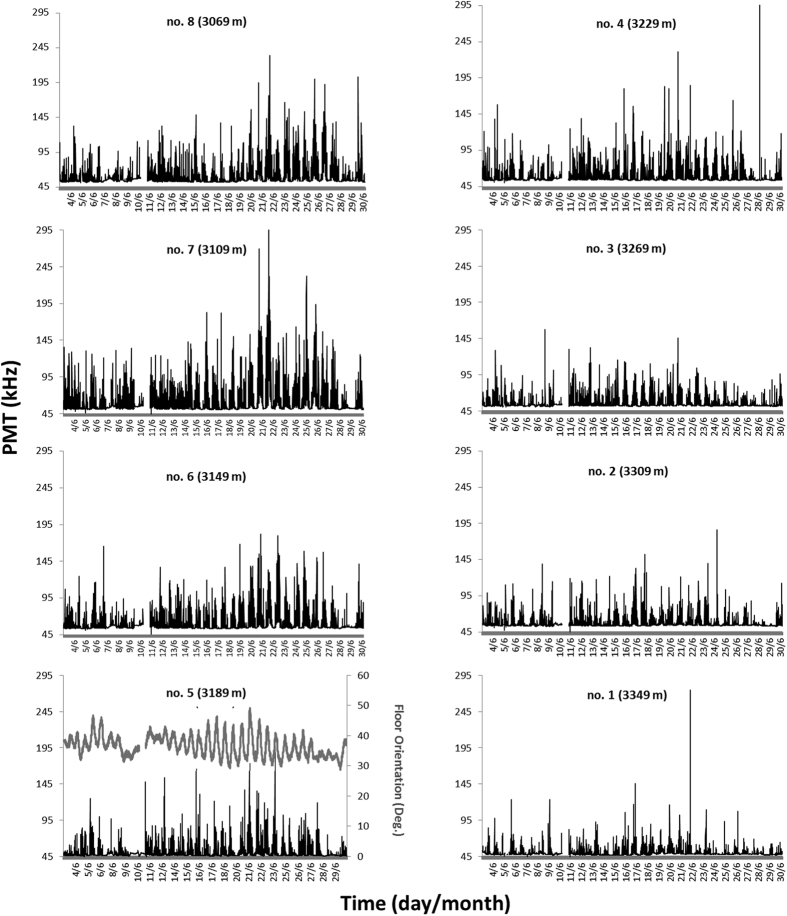
Time series of PMT readings for the 8 sampling floors of the telescope tower, as recorded during the entire testing period of June 2013. Floor no. 5 has been plotted together with its displacement time series, to underline differences in amplitude between the 1^st^ (3–16^th^ of June) and the 2^nd^ period (17–30^th^ of June). It should be noticed that the light emitted by ^40^K decay is around 45–50 kHz, so bioluminescence readings are above that background threshold. Short period gaps in data sets are due to failure in data acquisition. Also, the presence of sparse outlier peaking values, sometimes several orders of magnitude larger than most other data points, should be noted.

We obtained a total of 32,970 bioluminescence readings of 10 min at the 8 telescope floors. Time series for these readings have been reported at all floors in Fig. 1 (for ease of visualization the time stamp is 24-h). Marked rhythmic fluctuation patterns were visible at all floors, although overall bioluminescent activity appeared to increase in the second half of the testing period. Few and short gaps in data acquisition were reported, with sparse outliers. These spike values were neither eliminated nor smoothed by filtering (e.g. moving average) as they were considered true biological signals not produced by instrument malfunctioning. Background light emitted by ^40^K decay was around 50 kHz, except for floors no. 1 and 5 where a minimum of 45 kHz was measured.

**Figure 2:**
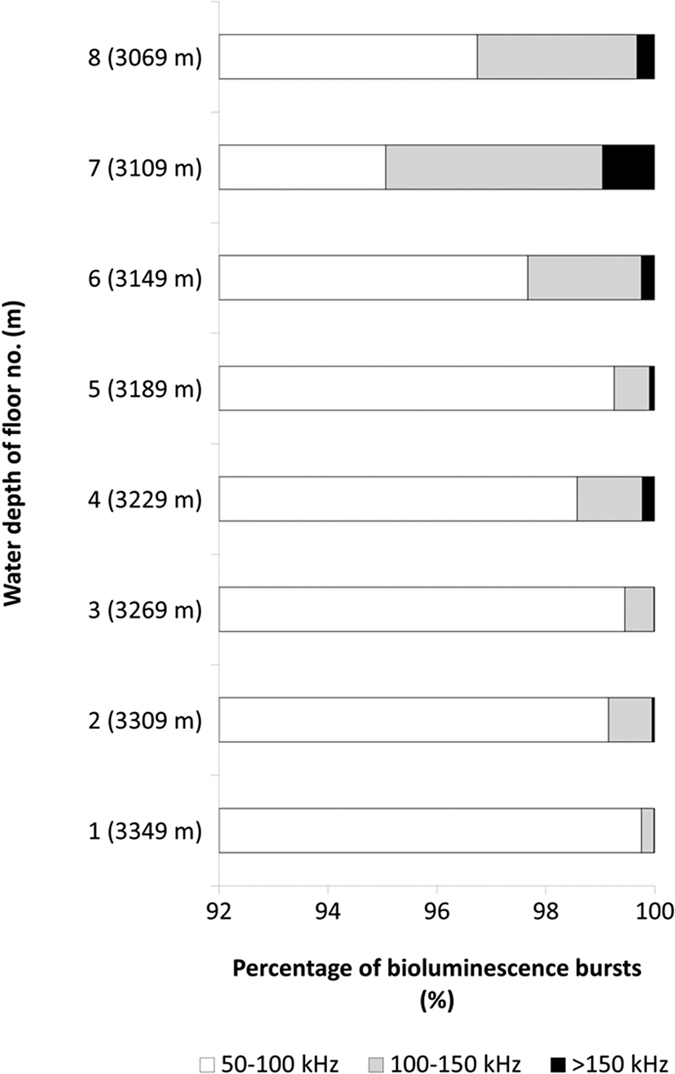
Percentages of bioluminescence bursts for each defined intensity range at each floor depth: white 50–100 (white); 100–150 (grey); and >150 kHz (black). Y-axis: numbers from 1 to 8 are floors and relative depth is reported in parenthesis.

The intensity of bioluminescence varied through floors as a function of depth (Fig. 2). Overall, a large majority of bursts (i.e. photons above a certain threshold that trigger a reading from the PMT) were reported within the 50–100 kHz range and a minority above 100 kHz. In particular, the presence of bursts within the 100–150 and >150 kHz ranges increased moving from floors close to the seabed, up within the water column.

A preliminary inspection of PMT time series (see Fig. 1) revealed differences in overall intensity from the first to the second half of the month, in agreement with changes in overall hydrodynamic regime (suggested by the increasing amplitude of floor displacement shown for floor no. 5). Accordingly, time series analysis was carried out by subdividing all biological and arm movement datasets into two halves of 13 days each, 3^rd^–16^th^ and 17^th^–30^th^ (16 and 17 full cycles of 1230 minutes, respectively); these were selected to have the maximum number of cycles for each sub-period while maintaining statistical representativeness. Evidence of the increase in burst intensity, from the 1^st^ to the 2^nd^ testing period, is further shown in Table 1, and confirmed by the reported amplitudes resulting from the Fourier analysis.

We detected a significant periodicity in bioluminescent intensity, corresponding to the rhythms of inertial currents at the latitude of the telescope site at all floors during the 1^st^ and the 2^nd^ testing period (periods between 19.1 and 20.8-h; Table 1). Rhythm strength as amplitude of the oscillation was generally low, with periodogram peak variance less than 20%. However, that periodicity progressively got stronger (as indicated by the overall increase in periodogram variance; see mean values in Table 1) when: *i.* moving from the first to the second half of the testing period; and *ii.* when moving away from the seafloor. In relation to this second finding, periodogram variance increased moving from deeper (i.e. lower) to shallower (i.e. higher) floors, as an indication of a depth-trend not only in intensity but also in the strength of the reported temporal patterning.

**Figure 3:**
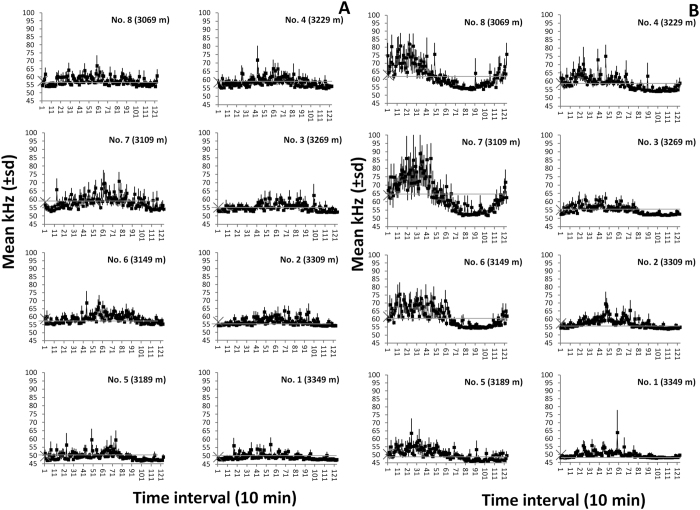
Waveform analysis outputs for data sets in bioluminescence for the 1^st^ (3–16^th^) and 2^nd^ (17–30^th^) period (**A** and **B**, respectively) of testing in June 2013, indicating the occurrence of temporally coherent peaking over the inertial day (i.e. time series subdivided into 123 sub-segments, as equivalent to 1230 min, according to periodogram analysis outputs). Waveforms are shown with associated standard errors. Horizontal dashed lines (starting from large crosses on the Y-axis) are the MESORs, defining peak temporal amplitudes, and also used as a proxy of overall mean bioluminescence (see Table 1).

In our results, we simultaneously assessed the composite changes in bioluminescence intensity both over time (at each floor) and depth (through floors). Bioluminescence rhythms appeared reduced in peak amplitude (hence periodicity strength) in the first monitoring period compared to the second monitoring period. In addition to this, the rhythms appeared stronger at the shallower floors. Clearly, these trends were combined and the results should be interpreted cautiously. Outputs from the waveform and Fourier analyses (Table 1 and Fig. 3) confirmed the periodogram outputs, evidencing the presence of mean patterns of fluctuations in bioluminescence at each telescope floor, with lower peak amplitudes in the first period (Fig. 3A) compared to the second one (Fig. 3B). In both monitoring periods, a dampening of bioluminescence occurred through the floors, becoming more evident in the second period.

**Figure 4:**
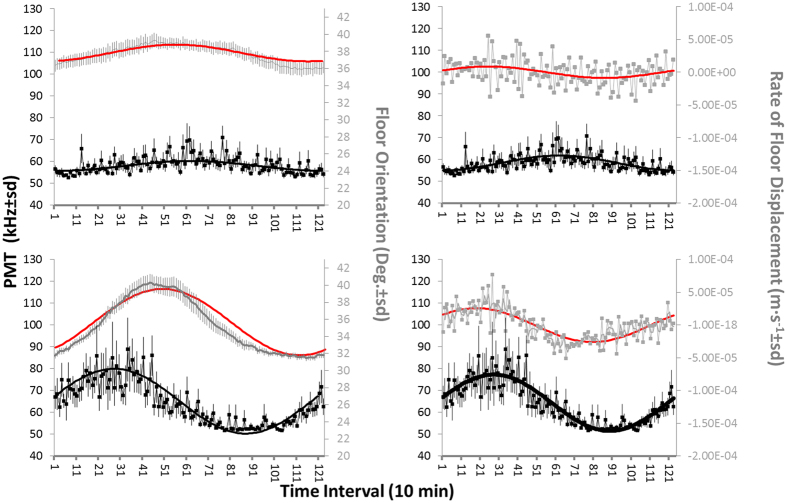
Waveform analysis output coupling of bioluminescence (black) phase (i.e. peak timing) with floor (grey) orientation (left) and rate of displacement (right). Data are shown with associated standard errors and Fourier curves (in black for PMT data and in red for floor orientation and rate of displacement). Following Fig. 4, we reported here PMT waveforms for floor no. 7 (depth: 3109 m) for the 1^st^ (3–16^th^) and 2^nd^ (17–30^th^) period (A and B, respectively) of June 2013. That analysis can be used as a proxy of the effect of currents on bioluminescence stimulation, based on the collision of pelagic animals against the telescope tower infrastructure.

To assess the likelihood of stimulation of bioluminescence by currents, outputs of biological and tower orientation waveform analysis were plotted together with Fourier components at the defined period (i.e. 1230 minutes), for floor no. 7 (Fig. 4). To perform a comparison, the rate of floor displacement, calculated using the floor orientation, was assumed to be a proxy for current speed. The corresponding continuous lines, derived from the Fourier analyses, basically reproduce the results of the waveform analysis: the main difference appears for the 1^st^ testing period, where the Fourier component of the rate of displacement (upward right panel in Fig. 4) shows a periodicity that did not appear in the waveform analysis. Furthermore, in the 1^st^ testing period, the PMT output appears in phase with the floor orientation, whereas in the 2^nd^ testing period it was the rate of displacement that noticeably overlapped with the bioluminescence signal. With respect to the angular data, it should be noted that the rotation of the structure is expected to be minimal as a result of its size, in line with the angular values shown in Fig. 4 (i.e. less than 5 degrees on the 1^st^ testing period and about 10 degrees on the 2^nd^).

**Figure 5:**
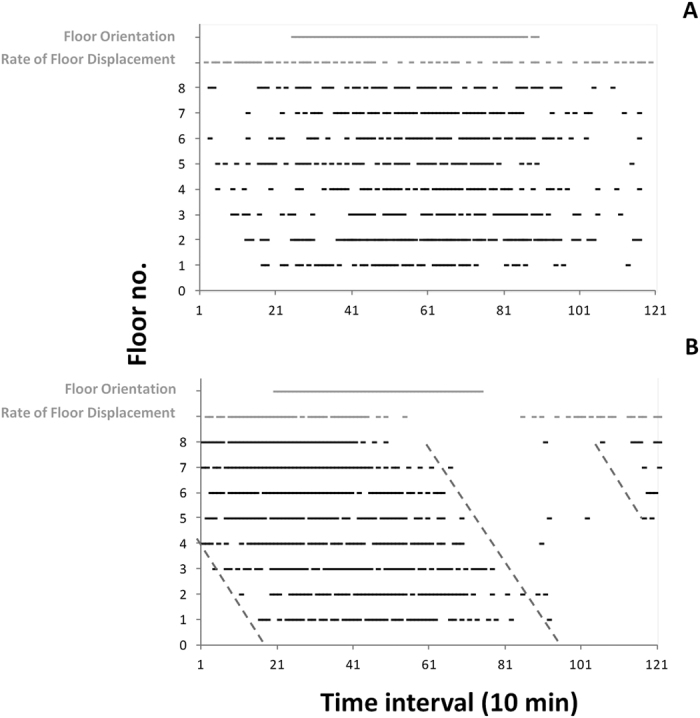
Phases’ relationship chart showing the amplitude of all waveform peaks for PMT (one per floor) and floor information (orientation and rate of displacement), as proxies of speed and angular direction, plotted together in relation to the inertial day-length (i.e. the time is in 10 min units, equivalent to time series sub-segments of 1230 min length). Oblique dashed grey lines are the visual fitting connecting waveform peak onsets and offsets though the floors.

Figure 5 shows the depth-oriented time shift in maximum bioluminescence (i.e. phase amplitudes, as computed by waveform analysis on PMT data at each floor; see Fig. 3) for the two testing periods. Peak onsets and offsets show a progressive depth delay along the tower axis, also confirmed by the phase values derived from the Fourier analysis (Table 1). The PMT antiphase peak is greater at shallower compared to deeper floors (i.e. maximum uncoupling at floor no. 8 and maximum phase synchronicity at floors no. 3 and 2). No similar conclusions can be made for the first testing period, where the rate of displacement shows a scattered distribution of PMT peaks (i.e. waveform phases) through the depth layers. Additionally, the Fourier-derived phases of bioluminescence fluctuate around the Fourier-derived phase of floor orientation (standard deviation of 60 min). This is in line with the progressive dampening of mean bioluminescence fluctuations (both intensity and strength) from shallower to deeper floors (see Fig. 2) and between testing periods A and B (see Fig. 1). The comparison of phases (both in the waveform and Fourier analyses) evidences how, in the second testing period, a temporal patterning in floor current-related displacement elicits a consequent temporal patterning in PMT recordings (see Fig. 5B).

## Discussion

In the present study, conducted in the deep central Mediterranean basin, inertial biological rhythms in bioluminescence appear as a response to inertial hydrodynamic cycles. We detected an inertial rhythm in the bioluminescent signals of abyssopelagic organisms, equivalent to a 20-h periodicity at the latitude of South Sicily^36^, onto background water emissions in agreement with results from Aiello *et al*.^37^. When tides occur under the form of weak bulge displacements, the much weaker inertial wind-driven currents propagate into the deep sea as descending whirlpools, detectable in current data. This phenomenon is thoroughly explained within the framework of our previous knowledge on deep-sea biological rhythms in the Mediterranean^3^,^5^,^8^.

The inertial-related periodicity reveals a coherent relationship between currents and bioluminescence. This suggests mechanical stimulation of bioluminescence, as organisms transported by flowing water collide with the deployed telescope infrastructure, resulting in the defensive emission of bioluminescent light^24^,^38^,^39^. The presence of temporally structured peaks and troughs in average overall bioluminescence and currents suggests that drifting organisms travel in discontinuous deep-sea undular vortices. These vortices consist of chains of inertially pulsating mesoscale cyclones/anticyclones advected by a background flow, created by huge water masses that slowly propagate (at speeds of approximately 2–3 cm/s) and can change direction, as has been described by Rubino *et al*.^35^. These appear to result from a delicate and complex balance between the forces acting in the basin and the topographic constraints, which require further investigation. Our time series analysis on bioluminescence data highlighted the occurrence of two composite patterns (see waveform analysis outputs in Fig. 3), supported by the occurrence of a temporal shift in peak timing as a function of depth (see the phases’ relationship in Fig. 5 and Table 1). Taken together, these results suggest that bioluminescent plankton are carried by the downward propagation of lens-like undular vortices, the force of which increased in our testing area during the second half of the monitoring period. Unfortunately, a precise identification of the origin of the described vortices is beyond the scope of this study and would require more precise oceanographic characterisation. However, it would be interesting to explore this aspect in future work, to determine whether the mechanism of generation is local or remote and whether it is of thermohaline origin.

Deep-sea currents can elicit a bioluminescent signal through mechanical stimulation^17^,^38^, as advected organisms collide with static structures of both man-made^40^ and natural^41^ origin. In a natural setting, bioluminescence is predicted to be stimulated by impinging currents at steep geomorphological structures such as outcrops, seamount flanks and tops, cliffs and escarpments^41^, as well as into canyons. We would therefore also expect a periodicity in the bioluminescent signal where these geomorphologies are exposed to rhythmic flow patterns. The ecological consequence of rhythmic variations in levels of bioluminescent activity at these sites is a matter for conjecture at this point with bioluminescence serving as an attractant or a feeding cue (e.g. refs 42 and 43) as well as a repellant (e.g. ref. 44). However, the prevalence of bioluminescence in the deep sea combined with its importance as a sensory cue would certainly indicate its potential as a synchronizing ecological cue to affect a rhythmic response in benthic communities. Likewise, temporal changes in bioluminescence produced at man-made structures could locally alter the behaviour of organisms, affecting their survival within the framework of predator-prey relationships. In view of our results we would recommend more extended measurement of bioluminescence and its interactions where water flow is interrupted, such as neutrino telescopes as well as complex seabed morphologies (especially seamounts and canyons), including in those areas where cabled observatory networks have been deployed and a further technological development is envisaged (e.g. the NEPTUNE network in Barkley canyon from the Ocean Network Canada, ONC; www.neptunecanada.ca).

Endogenous rhythms are those measured in laboratory controlled conditions, when the synchronizing environmental stimulus is removed (hence rhythms appear as “free-running” with a phase that progressively shifts over consecutive day cycles away from the time window driven by the absent external synchronizer)^45^. Constant condition protocols are therefore used to distinguish between endogenous (persistent, free-running) and exogenous (progressively fading out) rhythms. To our knowledge, no laboratory observations of deep-sea bioluminescent organisms exist, whose activity has been tracked over consecutive days in constant conditions (i.e. with light and current cycle deprivation). In the field, the most proximate conditions simulating constant laboratory darkness as a proxy of our deep-sea results would be the Arctic in winter^46^. Interesting field observations on 24-h based DVMs and bioluminescence patterning has been provided at this latitude and season. In our case, the bioluminescent patterning was not spontaneous but associated to currents and therefore its endogenous component is still elusive.

The question of the identity of the organisms producing the bioluminescence remains to be resolved. Bioluminescent organisms each have a characteristic light emission in terms of its spectrum, intensity, and kinetics^13^. Spectral and intensity data have been reported for several deep-sea pelagic organisms^18^,^47^. However, the intensity and kinetics of the light produced is also dependent on the type and strength of the stimulus (e.g. refs 48 and 49, and in the absence of a standardized mode of stimulus, to try and identify the organisms in question from the PMT data alone is not likely to be possible. However, our results show that a greater proportion of organisms with brighter bioluminescent emissions were likely present higher within the water column than near the seafloor. This may result from different communities present at different depths. The presence of changes in community throughout the benthopelagic layer is well documented (refs 50 and 51 and literature herein). Deep-sea zooplankton with the brightest emissions include some species of copepod and decapod, as well as gelatinous organisms, including scyphozoans, siphonophores, and pyrosomes (reviewed in ref. 18, and these may have been present in greater concentrations at increasing altitude above the seafloor. In the future, additional information could be obtained from neutrino telescopes about individual bioluminescent flashes and their probable source if they were instrumented with cameras capable of imaging individual flashes, such as the ICDeep^50^, LuSEApher^52^ or sCMOS^53^ cameras.

Continual monitoring of deep-sea bioluminescence by neutrino telescopes at various sites within the Mediterranean Sea has the potential to make important spatial and temporal comparisons of productivity and its export to the deep sea. Supporting the findings in the present study, tidal rhythms were also observed at a deep telescope site on the coast of Greece (Pylos)^39^, which were not detected at Capo Passero. At the western Mediterranean ANTARES, light production is generally higher by an order of magnitude than at the NEMO site (i.e. approx. 10^2^ here *versus* 10^3^ kHz, as reported by 24 for the same month). The difference in bioluminescence signal between the two sites is similarly reflected in bioluminescence data collected in the deep sea using low light video cameras^17^,^38^,^54^, most likely resulting from a combination of differences in depth as well as higher levels of productivity in the western Mediterranean, fueling a greater abundance and biomass of deep-sea organisms^55^. Seasonal variations have been reported from the ANTARES site with observations of strong bioluminescence signals, generated from bioluminescent bacteria and other marine organisms, associated with deep-water formation in winter^24^,^26^. Seasonal variations in the abundance of bioluminescent zooplankton have been observed at the Capo Passero site from video data, with a deep peak forming in spring^54^. This could be attributed to the arrival of phytodetritus to the deep sea with a delay of two-three months^55^,^56^ with respect to the peak of primary production at the surface, typical of this area in late winter (e.g. see February-March satellite data from http://giovanni.gsfc.nasa.gov/giovanni/). The greater food availability for deep-sea organisms likely enhances zooplankton biomass, possibly resulting in the increase in bioluminescent activity observed in the latter half of June in the present study.

Cabled observatory networks including branching neutrino telescopes open an important window for real-time, continuous, and long-lasting observation of the abyss^57^,^58^. Further work along these lines can be used to develop the use of biological data to support oceanographic studies and *vice versa*: the detection of bioluminescence rhythms can reveal the existence of deep-sea mesoscale features and their variability. In this context, automation of data processing per our multiple step analysis protocol inspired by chronobiology (i.e. periodogram, waveform and phase integrated chart analyses) are of relevance for the real-time and continuous monitoring of abyssal bioluminescence diel and seasonal dynamics.

## Materials and Methods

### The NEMO Phase 2 prototype

Cherenkov neutrino telescopes are research infrastructures whose primary goal is the detection of high-energy neutrinos of astrophysical origin, observable in the vast darkness of the deep sea^59^. An underwater neutrino telescope is an array of several thousand PMTs and oceanographic equipment (e.g. hydrophones and oceanographic sensors) displaced over a volume of water of about a cubic kilometre. Power and data transport is obtained using a network of electro-optical cables, connected to a main cable landing on-shore^60^. Thanks to their remote, real-time and multiparametric measurement capability, these structures can be opened to more multidisciplinary research and presently serve a diversified community of end-users such as oceanographers, biologists, and geophysicists^61^.

**Figure 6:**
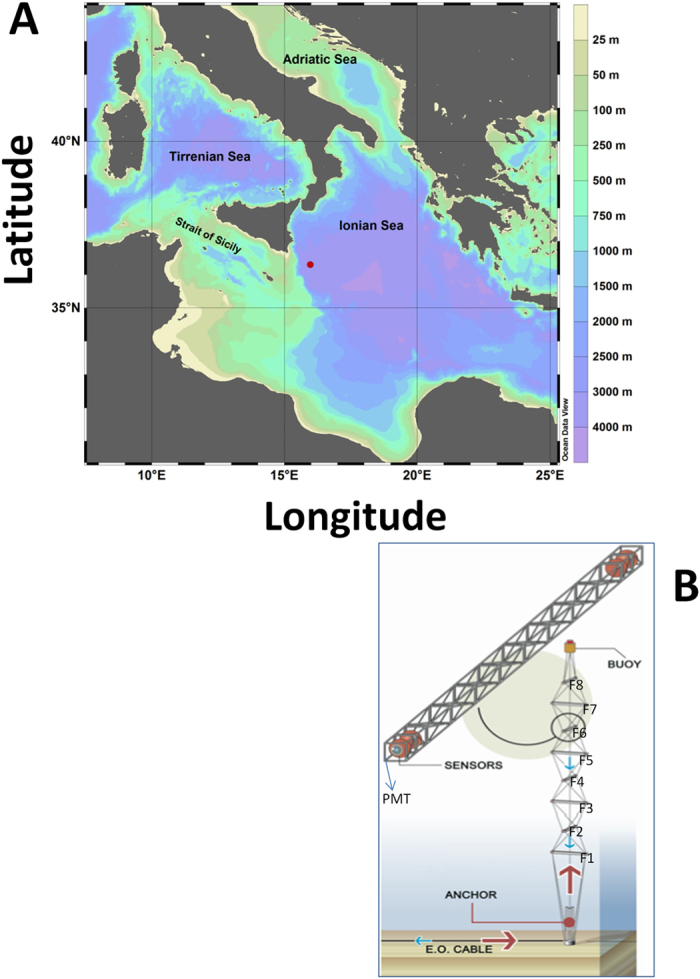
Location (red dot) and bathymetry (**A**) of the Capo Passero Site, hosting the KM3NeT-Italia neutrino telescope, in the Central Mediterranean (the red dot indicates the coordinates of the prototype NEMO Phase 2 tower, Ionian Sea South-East of Sicily at 36° 16′N, 16° 06′E). The map was produced with Ocean Data View, ODV 4.7.8^74^. Telescope tower structure (**B**) reporting the eight-floor (F) positioning and the arm holding the 2 photomultipliers (PMT) at its extremes. That drawing was produced by the KM3-Net Italy Consortium.

A prototype detection unit, the NEMO Phase 2 tower^37^,^62^ was deployed at 3450 m depth, about 90 km off-shore Capo Passero in March 2013 (Ionian Sea; Fig. 6A,B), within the activities of the NEMO (Neutrino Mediterranean Observatory) collaboration. The tower contains 32 optical modules, each comprised of a glass sphere containing a Hamamatsu R708110 Photo-Multiplier Tube (PMT), with single photon sensitivity within the 400–700 nm wavelength range, and its readout electronics^63^. The four optical modules are deployed at both extremities of an 8 m-long horizontal arm, two looking horizontally, two downward oriented, to optimise reconstruction of horizontal and up-going neutrino tracks. Here, we used data from pairs of horizontal-looking optical sensors for each floor. The tower rises 420 metres above the bottom and contains 8 arms representing measuring floors, separated by 40 m (i.e., from average CTD readings, 3349, 3309, 3269, 3229, 3189, 3149, 3109, and finally 3069 m depth, see Fig. 6B). PMT readout electronics provides two kinds of data: a) pulse shape of detected photon hits (signals exceeding 0.3 single photoelectrons threshold) sampled with 5 ns bin; b) average rate of photon hits recorded in 1 ms every second. The latter are used in the present work.

The PMT counting rate of an undersea neutrino detector is affected by constant background environmental light. The decay of radioactive elements in seawater, mainly potassium 40 (^40^K), causes a steady production of photons; Cherenkov radiation^64^,^65^. The emission spectra of most bioluminescent deep-sea organisms are blue (centered around 475 nm^13^, which also falls within the sensitivity of the PMTs, therefore also adding to the optical background in the data. Bioluminescent bacteria produce a steady glow when cell density exceeds a critical level^66^. When present at sufficient densities, bacterial bioluminescence adds to the Cherenkov radiation to produce a constant baseline (background) counting rate in the PMT data. Sharp peaks often several orders of magnitude higher than the steady baseline rate, are also found in the PMT data^67^. These peaks result from collisions between bioluminescent organisms and the telescope structure and can be correlated with current velocity^64^.

### Data collection

We focused on bioluminescence readings for the month of June 2013 (from 03/06 at 6:57 to 30/06 at 23:57), as detected separately by PMT pairs at the 8 floors of the NEMO tower prototype (GPS coordinates: N 36° 17′48.12″, E 15° 58′45.06″, 3450 m depth). Light intensity readings obtained by both PMTs (that is the optical rate measured in 1 ms every second) were averaged together at 10 min frequency. We carried out time series pre-processing by eliminating acquisition gaps with a moving average, calculated using a variable number of precedent and successive values depending on gap duration. This time series reconstruction (in 10 min bins) did not alter the detection of significant periodicities at diel scale (i.e. encompassing tidal, inertial and day-night related rhythms) as it was carried out well below the Nyquist frequency law restrictions.

The angular movement of the tower arms has been used as a proxy for water flow direction and speed changes. The arm orientation was measured *via* the electronic compass installed at the fifth floor, and cross-checked with data of the acoustic positioning system: acoustic transceivers were anchored at fixed positions on the sea floor, whereas acoustic receivers (i.e. hydrophones) were fixed along the tower. The hydrophone positions were determined by triangulation, using the travel times of acoustic signals between emitters and receivers^62^,^68^. The rate of displacement of the arm orientation was also calculated.

### Data analysis

Although the burst rate cannot be aligned with specific groups of organisms, a comparison was made of the bioluminescence data with respect to burst intensity to assess broad differences in the bioluminescent community at different altitudes above the seafloor. Bioluminescent emission can vary according to the strength of the stimulus and the degree of exhaustion of an individual specimen^18^,^49^,^69^, however a reasonable comparison of bioluminescent intensity can be made assuming similar water and current conditions at each tier of the tower. So PMT data were subdivided into ranges of intensity for each floor. Bioluminescent bursts were counted and grouped into 3 arbitrary intensity categories: 50–100, 100–150, and > 150 kHz. Values were then transformed into percentages and visually compared through the different sampling depths, to give an indication of the distribution of bioluminescent emissions with respect to intensity.

The occurrence of significant periodicities in PMT time series (i.e. all detections above 45 kHz), tower angular movements and displacements was assessed by using the chi-square periodogram procedure^70^, with El Temps package (www.el-temps.com). The periodogram analysis is an extension of the Buys-Ballot Table (Williams and Naylor, 1978^71^. When a periodogram analysis is performed, the periodicity of the inherent time series is unknown. Thus, the period is screened by testing for a range of wavelengths selected to cover all rhythms of interest (e.g. tidal, inertial, and day-night related). Periodicities were screened within the interval 400–1600 min. In the periodogram output plots, the highest peak exceeding the significant threshold (*p-value* < 0.05) represents the maximum percentage of total data variance explained by the inherent dominant periodicity.

A peak is defined as a single increase in the time series fluctuation over a cycle time window. When this fluctuation is averaged over several cycles in order to obtain an approximated timing of time series increase, a phase can be computed *via* waveform analysis. A waveform analysis^72^ was carried out on biological and floor orientation data, by subdividing time series on the length of the dominant inertial periodicity *T* at the latitude ϕ of the study area, i.e. between 20 and 21-h^62^. Briefly, we subdivided time series into sub datasets of 20.5 h length, equivalent to 1230 min. Then a consensus curve (i.e. the waveform) was obtained by averaging values within all subset *per* corresponding 10 min bins. Phase duration and timing for bioluminescence were statistically assessed using the Midline Estimating Statistic Of Rhythm (MESOR)^73^ all waveform values were re-averaged and the resulting mean represented as a horizontal threshold line on the waveform plots. This threshold was used to identify waveform peaks as temporal amplitude of significant increments (i.e. the phase duration^74^. To further validate our methodology and corroborate the waveform using a different approach, an independent Fast Fourier Transform analysis (FFT) was also conducted on both biological and angular data, for the 1^st^ and the 2^nd^ testing period to produce corresponding results.

Outputs for waveform analysis of bioluminescence and floor orientation data were plotted together to assess the phase relationship between bioluminescence and water flow as a proxy of cause-effect relationships (i.e. water flowing against the telescope structure causing mechanical stimulation of bioluminescence in advected organisms). To do so, we presented data for the floor at which the largest biological and angular amplitude fluctuations were reported. The changes in peak timing through floors for bioluminescence rhythms were evidenced by plotting all waveform phases into a common plot^57^. In that chart, the temporal amplitude of each waveform (i.e. the phase made by all values above the MESOR; see above), was shown as a continuous horizontal line. To evidence any depth-related trend in the rhythms of peak timing, used as a proxy of vertical movement and downward propagating whirlpools, different phase durations were plotted together for all floors. Then, the “eye-fitting method” generally in use in chronobiology^45^, was applied to visually identify a depth-shift in phases. An observer marked eye-fitted straight lines, connecting the onset and offset of waveform phases through floors. This fitting approach is used to predict the timing of a significant increase in a rhythm (i.e. its peak), based on its temporal pattering during previous 24-h cycles. This is very useful when an observation must be gathered in the field or an experimental treatment must be administered in the laboratory at a certain moment of a rhythm, without a real-time knowledge of its fluctuation state. Here, we adapted this method to identify bioluminescence phase shifts through depth, as temporal difference in activity onsets and offsets at all floors.

## Additional Information

**How to cite this article:** Aguzzi, J. *et al*. Inertial bioluminescence rhythms at the Capo Passero (KM3NeT-Italia) site, Central Mediterranean Sea. *Sci. Rep.* **7**, 44938; doi: 10.1038/srep44938 (2017).

**Publisher's note:** Springer Nature remains neutral with regard to jurisdictional claims in published maps and institutional affiliations.

## Figures and Tables

**Table 1:** PMTs bioluminescence reading performance per floor and time series analysis outputs for the 2 consecutive periods in June 2013 (3^rd^–16^th^ and 17^th^–30^th^).

PMT								
Testing Period	Floor	MESOR		Significant Periodicity			Fourier analysis	
	(depth, m)	Mean (kHz)	sd	h (centimes)	h (min)	Variance (%)	Amplitude (kHz)	Phase (min)
**1st**	8 (3069)	57.4	2.6	—	Arr.	—	0.8	−524
	7 (3109)	58.4	3.8	20.8	1250	10	1.7	−615
	6 (3149)	58.7	2.8	20.7	1240	11.1	1.3	−625
	5 (3189)	49.7	2.4	20.7	1240	11.2	0.9	−517
	4 (3229)	58.5	2.8	19.1	1145	9.2	1.1	−566
	3 (3269)	55.2	2.3	—	Arr.	—	0.9	−619
	2 (3309)	56.5	2.2	20.8	1250	9.5	0.9	−601
	1 (3349)	49.4	1.7	20.6	1235	9.8	0.7	−479
**Meansd**				20.4	1226.7	10.1	1.0	−568
				12	40.5	0.8	0.3	56
**2nd**	8 (3069)	62.8	7.5	20.2	1210	19.2	4.6	−197
	7 (3109)	64.1	9.7	20.3	1220	17.7	6.5	−268
	6 (3149)	61.6	5.9	20	1200	17.5	3.6	−286
	5 (3189)	50.4	3.4	20	1200	12.8	1.9	−307
	4 (3229)	58.9	4.2	20	1200	12.2	2.2	−313
	3 (3269)	55.2	2.7	20.3	1220	14.2	1.4	−397
	2 (3309)	57.3	3	20.2	1210	12.9	1.6	−487
	1 (3349)	49.8	2.2	20.3	1215	9.7	1	−452
**Meansd**				20.2	1209.4	14.5	2.9	−338
				0.1	8.6	3.3	1.9	98
**Floor Orientation**								
**Testing Period**		**MESOR**		**Significant Periodicity**			**Fourier analysis**	
		**Mean (deg.)**	**sd**	**h (centimes)**	**h (min)**	**Var. (%)**	**Amplitude (deg.)**	**Phase (min)**
**1st**		37.7	1.1	19.1	1145	24.4	0.7	−543
**2nd**		35.2	3.1	20.3	1215	59.1	2.2	−480

Significant periodicities (hours in centimes and minutes) are reported with the percentage of data variance (i.e. periodogram peak height as a measure of rhythm strength). MESOR (and its standard deviation, sd) is also reported as a proxy of overall bioluminescence for each waveform analysis at each floor. Amplitude and phase values from the Fourier analysis are also shown. Arr. stands for arrhythmic values.
